# Supplementary material for: Synchrony of Bird Migration with Global Dispersal of Avian Influenza Reveals Exposed Bird Orders
Source: Nat Commun. 2024 Feb 6;15:1126. doi: 10.1038/s41467-024-45462-1 (PMC10847442; doi:10.1038/s41467-024-45462-1)
Supplement: Supplementary file 1 — Supplementary Information [file 41467_2024_45462_MOESM1_ESM.pdf]

Supplementary Materials for

Synchrony of Bird Migration with Global Dispersal of Avian Influenza Reveals Exposed Bird Orders

Yang et al., 2023

This PDF file includes:

Figure. S1 to S8
Table S1, S2, S3, S4
References

Other Supplementary Material for this manuscript includes the following:

Supplementary dataset 1, 2, 3
Code and scripts on the GitHub repository
(https://github.com/kikiyang/HPAI_Bird_world)

Table S1 Independent variables of the species distribution model of wild birds.

Variables	Source
Normalized Difference Vegetation Index monthly mean	Pinzon et al(2014) [1] (https://iridl.ldeo.columbia.edu/SOURCES/.NASA/.ARC/.ECOCAST/.GIMMS/.NDVI3g/.v1p0/index.html?Set-Language=en)
Evergreen Deciduous Needleleaf Trees	Tuanmu and Jetz (2014) [2] (www.earthenv.org/landcover)
Evergreen Broadleaf Trees	Tuanmu and Jetz (2014) [2]
Deciduous Broadleaf Trees	Tuanmu and Jetz (2014) [2]
Mixed/Other Trees	Tuanmu and Jetz (2014) [2]
Shrubs	Tuanmu and Jetz (2014) [2]
Herbaceous Vegetation	Tuanmu and Jetz (2014) [2]
Cultivated and Managed Vegetation	Tuanmu and Jetz (2014) [2]
Regularly Flooded Vegetation	Tuanmu and Jetz (2014) [2]
Urban/Built-up	Tuanmu and Jetz (2014) [2]
Snow-Ice	Tuanmu and Jetz (2014) [2]
Barren	Tuanmu and Jetz (2014) [2]
Open Water	Tuanmu and Jetz (2014) [2]
Mean Temperature	WorldClim 2.1[3] (www.worldclim.com/version2)
Wind Speed	WorldClim 2.1[3]
Total Precipitation	WorldClim 2.1[3]
Elevation	SRTM elevation data[4–8] (https://doi.org/10.5069/G9445JDF)

Table S2 Generalized linear model results (controlling for viral sample size)

Predictor	Inclusion*	BF†	cEffect (95% HPD)‡
**Clade 2.3.2.1**			
Viral sample size (O)	1	infinite	-1.52 (-1.86, -1.20)
Poultry trade	0.038	0.15	0.07 (-0.34, 0.37)
Bird migration	0.29	1.55	1.19 (-2.80, 4.23)
**Clade 2.3.4.4 (2010-2017)**			
Viral sample size (O)	1	infinite	-1.49 (-1.80, -1.14)
Poultry trade	0.93	54	0.71 (0.25, 1.25)
Bird migration	0.99	267	2.68 (1.27, 4.11)
**Clade 2.3.4.4 (2018-2023)**			
Viral sample size (O)	1	infinite	-0.91 (-1.15, -0.68)
Poultry trade	0.24	1.18	0.25 (0.012, 0.54)
Bird migration	1	infinite	4.72 (3.66, 6.27)

*Inclusion, probability that the predictor was included in the model.

†BF, Bayes factor.

‡cEffect, conditional effect size, which represents the estimate of the coefficient conditional on the predictor being included in the model. Both the mean and the 95% highest posterior density credible interval (95% CI) of the conditional effect size are reported.

Table S3 Statistically significant associated bird order distribution at origin/destination location and virus lineage movements, adjusted for multiple comparison. Block bootstrapping was used to calculate confidence intervals and two-tailed *p* values. See details in Methods 4.4.

Virus lineage movement (origin-destination)	Bird order distribution at origin	Mean Pearson's correlation coefficient* (97.5% CI)	*P* value
**Clade 2.3.2.1**	\	\	\
**Clade 2.3.4.4 (2010-2017)**			
Japan/Korea – Southern China	Anseriformes (origin)	-0.26 (-0.51, -0.13)	$2.78*10^{-5}$
Southern China – Southeast Asia	Accipitriformes (origin)	-0.43 (-0.65, -0.20)	$5.56*10^{-5}$
**Clade 2.3.4.4 (2018-2023)**			
Europe - Africa	Ciconiiformes (origin)	-0.47 (-0.65, -0.29)	$3.58*10^{-5}$
Western Asia - Africa	Passeriformes (destination)	0.52 (0.33, 0.79)	$5.38*10^{-5}$
Japan/Korea - Russia	Pelecaniformes (origin)	-0.32 (-0.46, -0.03)	$8.96*10^{-5}$

* Mean correlation coefficient, mean of the correlation coefficient distribution of the bootstrapped samples.

Table S4 Implications of the statistical association between bird order distribution probability and virus lineage movements

Rule #	Birds at origin/destination	Negative/Positive correlation	Implication: Role of the bird order in virus dispersal
1	Origin	Negative	Participating in virus lineage movements on the route if (4) is also true; being exposed to viruses when the birds migrate from the origin location
2	Origin	Positive	Not spreader of the viruses at the origin; being exposed to viruses when they migrate to the origin location
3	Destination	Negative	Not spreader of the viruses at the destination; being exposed to viruses when they migrate from the destination location
4	Destination	Positive	Spreader of the route if (1) is also true; being exposed to viruses when they migrate to the destination location

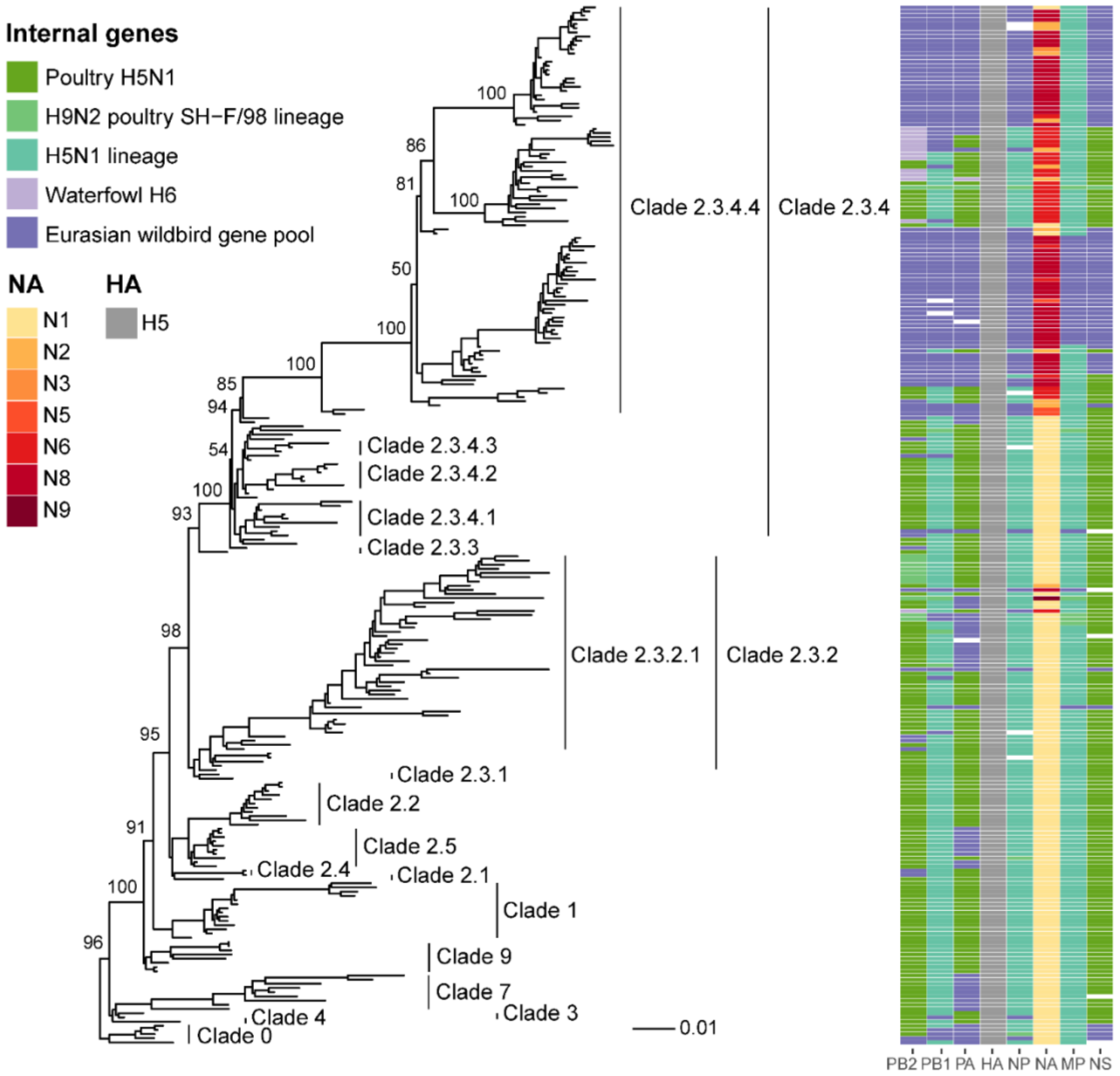

Figure S1. Maximum likelihood tree of hemagglutinin (HA) genes and genotypes of all gene segments of H5 subtype avian influenza A viruses. Poultry sources of the internal genes are coloured in green and wild bird sources are in purple. Yellow to dark red colours indicates different types of neuraminidase (NA) gene.

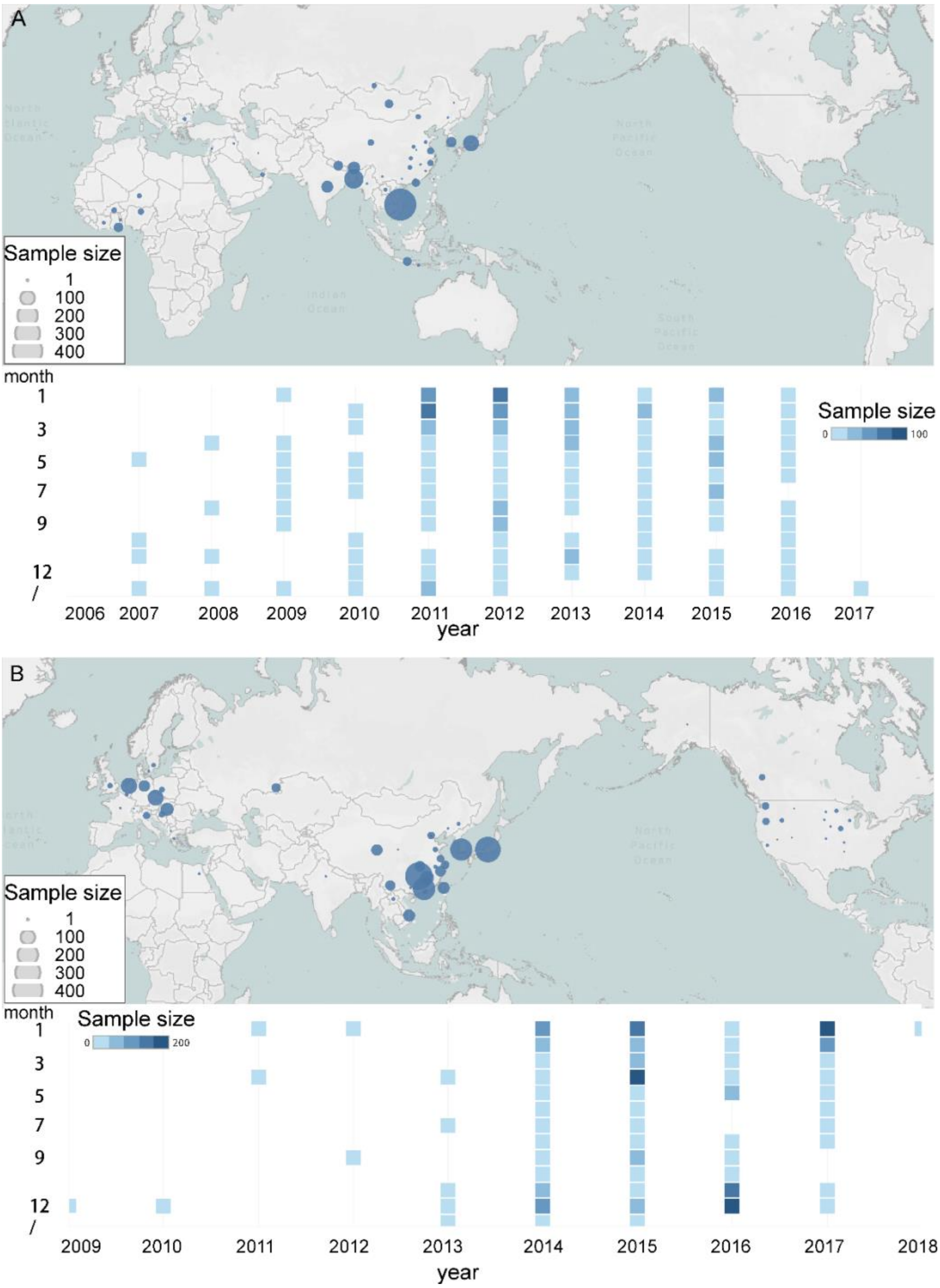

Figure S2. Spatial-temporal distribution of HA sequences of Clade 2.3.2.1 ***(A)*** and Clade 2.3.4.4 (2007-2017) ***(B)***. The light to dark blue colours represent the number of the sequences sampled in a month.

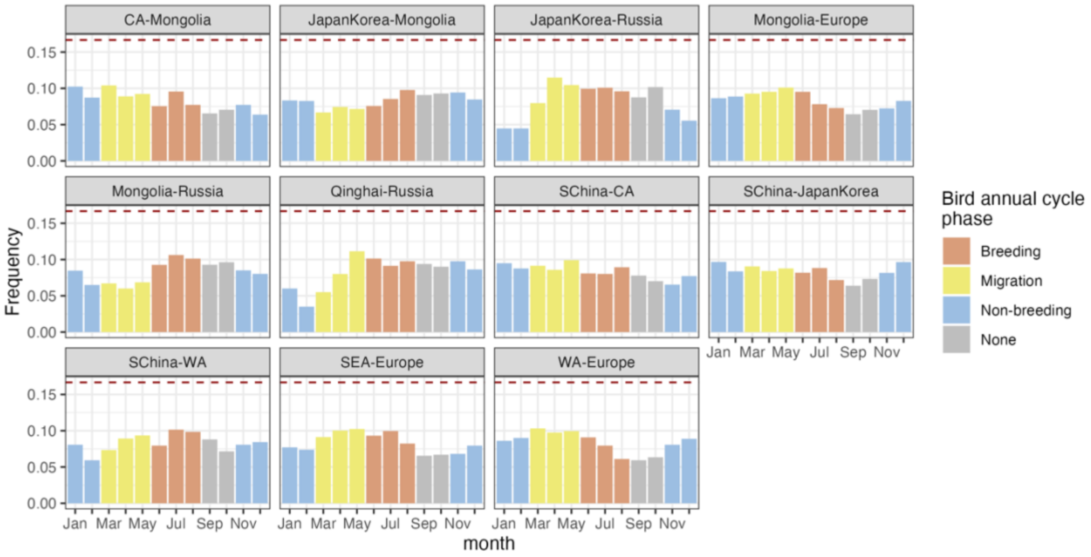

Figure S3.1 Frequency density distribution of Markov jump events of clade 2.3.2.1 each month through a year from year from southern to northern regions. We consider a peak of virus lineage movement when the frequency > 0.167 (=2*0.083, if the frequency is evenly distribution across months, monthly frequency should be 0.083). The frequencies are summarized over 4501 posterior trees.

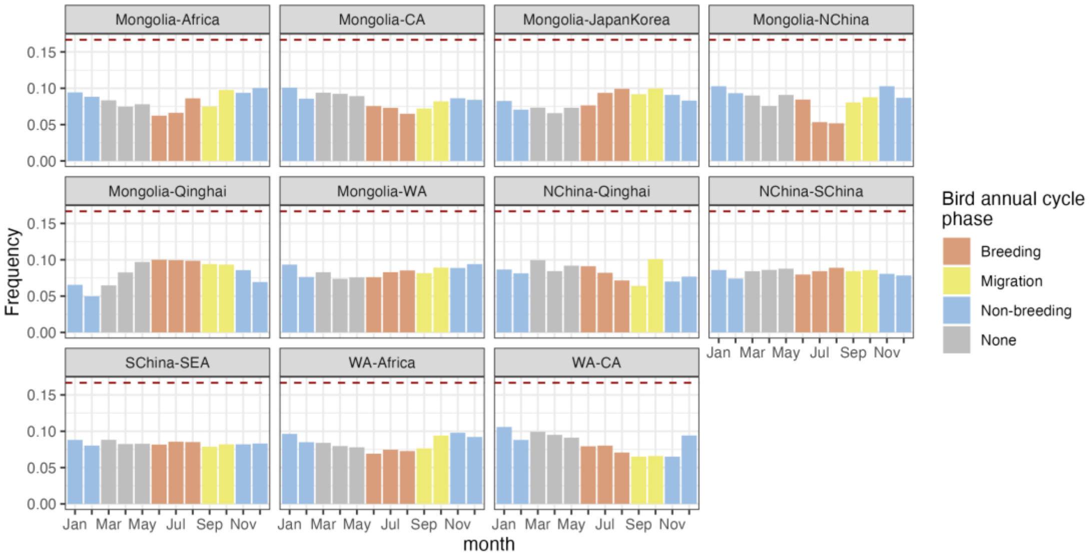

Figure S3.2 Frequency density distribution of Markov jump events of clade 2.3.2.1 each month through a year from year from northern to southern regions. We consider a peak of virus lineage movement when the frequency > 0.167 (=2*0.083, if the frequency is evenly distribution across months, monthly frequency should be 0.083). The frequencies are summarized over 4501 posterior trees.

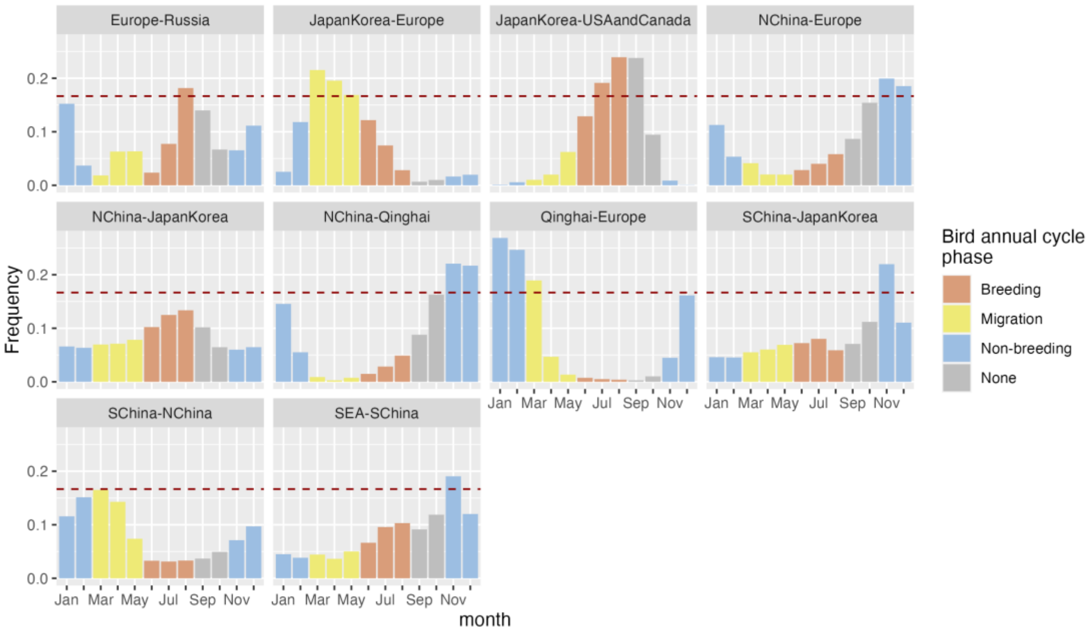

Figure S3.3 Frequency density distribution of Markov jump events of clade 2.3.4.4 (2010-2017) each month through a year from southern to northern regions. We consider a peak of virus lineage movement when the frequency > 0.167 (=2*0.083, if the frequency is evenly distribution across months, monthly frequency should be 0.083). The frequencies are summarized over 81000 posterior trees.

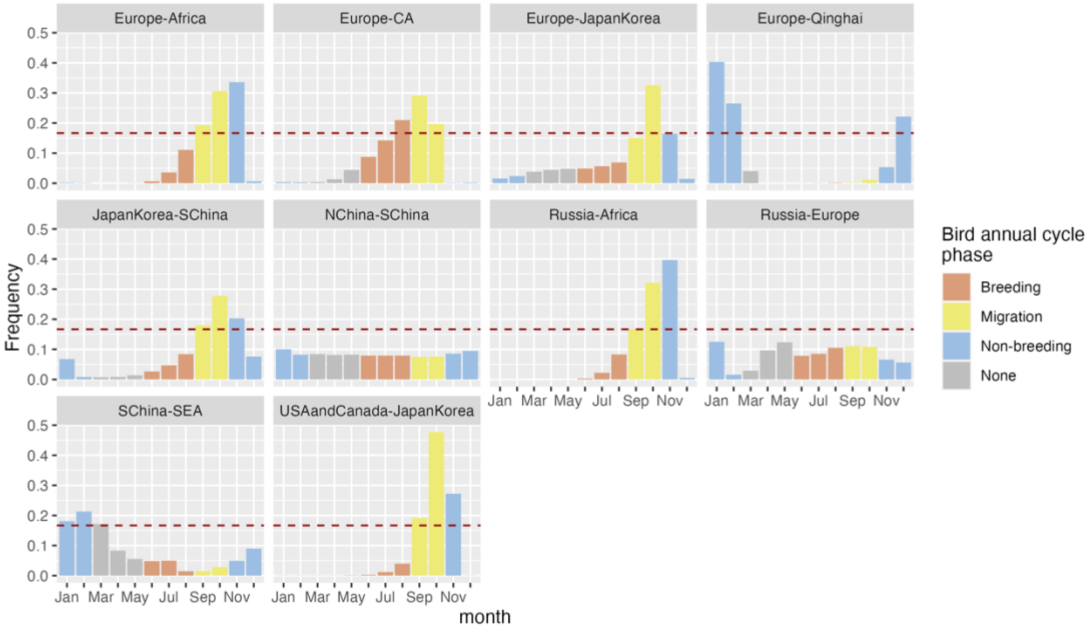

Figure S3.4 Frequency density distribution of Markov jump events of clade 2.3.4.4 (2010-2017) each month through a year from year from northern to southern regions. We consider a peak of virus lineage movement when the frequency > 0.167 (=2*0.083, if the frequency is evenly distribution across months, monthly frequency should be 0.083). The frequencies are summarized over 81000 posterior trees.

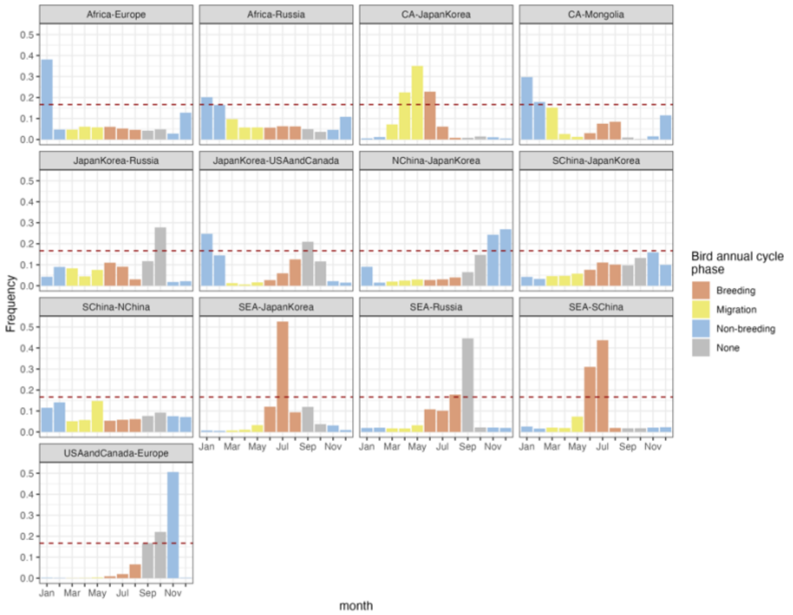

Figure S3.5 Frequency density distribution of Markov jump events of clade 2.3.4.4 (from 2018 to 2023) each month through a year from year from southern to northern regions. We consider a peak of virus lineage movement when the frequency > 0.167 (=2*0.083, if the frequency is evenly distribution across months, monthly frequency should be 0.083). The frequencies are summarized over 5631 posterior trees.

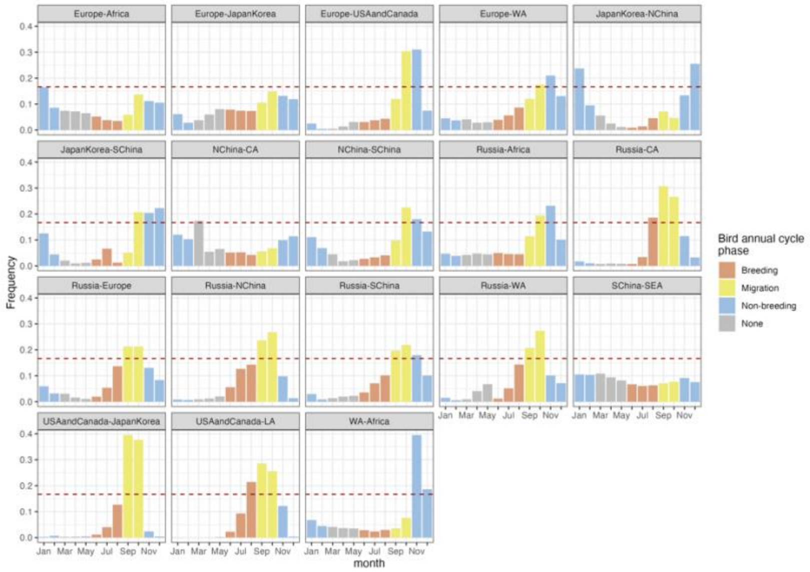

Figure S3.6 Frequency density distribution of Markov jump events of clade 2.3.4.4 (from 2018 to 2023) each month through a year from year from northern to southern regions. We consider a peak of virus lineage movement when the frequency > 0.167 (=2*0.083, if the frequency is evenly distribution across months, monthly frequency should be 0.083). The frequencies are summarized over 5631 posterior trees.

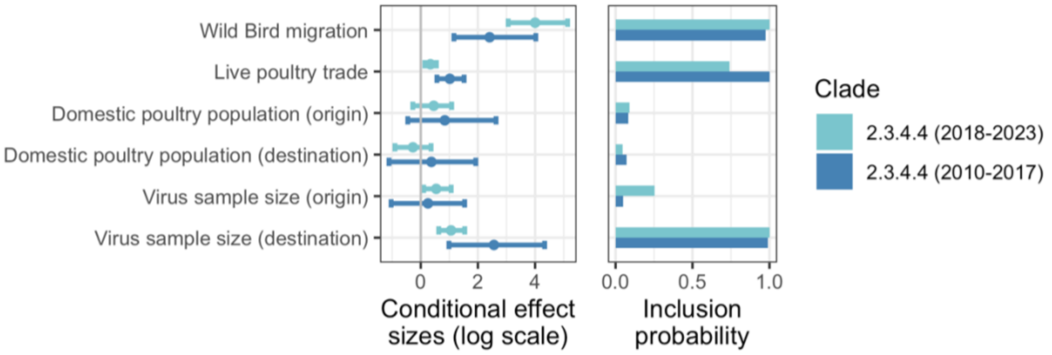

Figure S4. Contributions of predictors to worldwide diffusion of H5N1 clade 2.3.4.4 inferred from HA genes by GLM-extended Bayesian phylogeographic inference with heterogeneous evolutionary processes through time (2010-2017, 2018-2023). The conditional effect size of each predictor is presented as mean values with credible intervals of the GLM coefficients on a log scale. The phylogeographic GLM estimates were obtained from n=1845, 1844, and 1163 viral sequences sampled over 12, 10, and 11 locations for clade 2.3.4.4 (2018-2023), 2.3.4.4 (2010-2017), and 2.3.2.1, respectively.

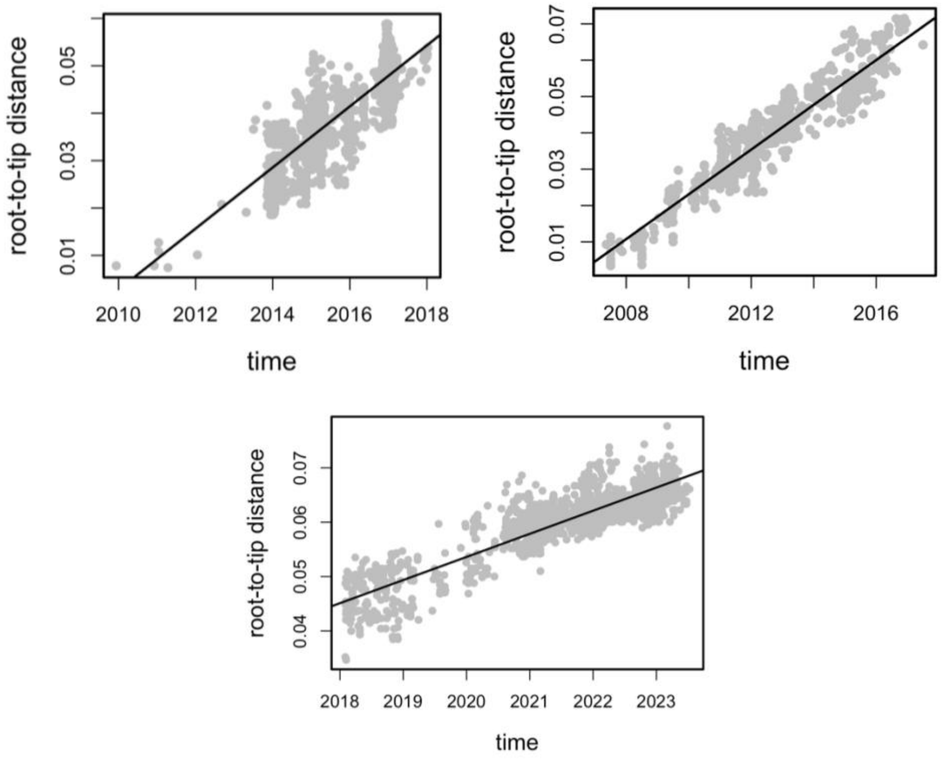

Figure S5. Strong temporal signal tested in TempEst of HA genes of HPAIV H5 clade 2.3.4.4 before 2018 (R squared=0.67, top left panel), that after 2018 (R squared=0.78, bottom panel) and clade 2.3.2.1 (R squared=0.89, top right panel). We used the final dataset of 1844 HA sequences of clade 2.3.4.4 (2007-2017), 1845 sequences of clade 2.3.4.4 (2018-2023), and 1163 HA sequences of clade 2.3.2.1.

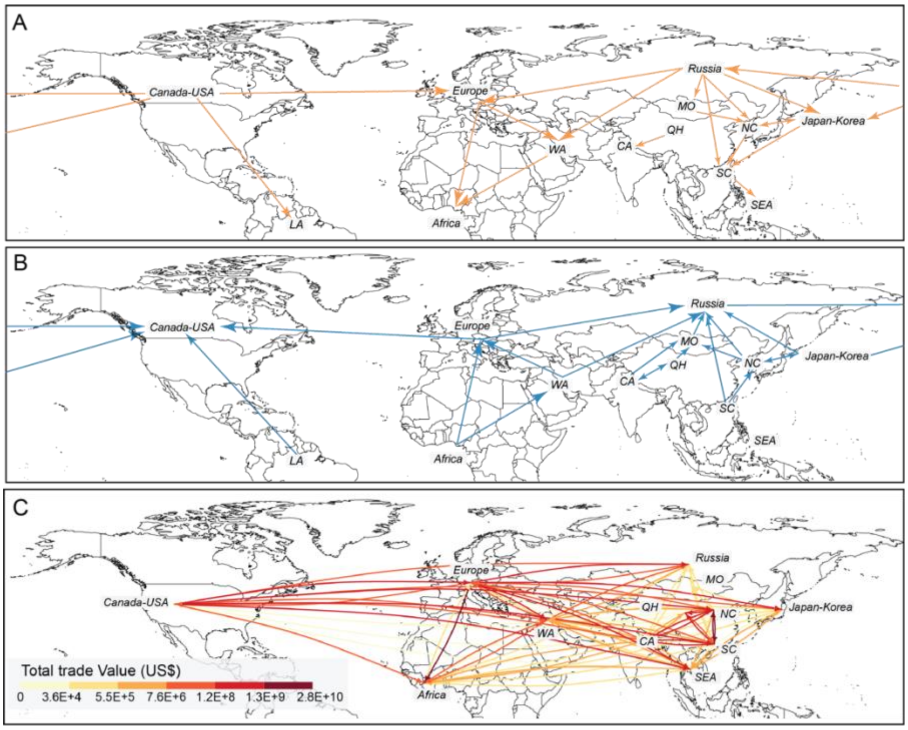

Figure S6 Bird migration network and live poultry trade network. (A) Northern Hemisphere fall season and (B) Northern Hemisphere spring season, where directed non-weighted edges represent the regions are connected by bird migration based on publicly-seen data on Movebank map, and (C) live poultry trade network, where directed weighted edges represent poultry trade value. NChina/NC: North China; SChina/SC: South China; SEA: South-East Asia; CA: Central Asia; QH: Qinghai; MO: Mongolia; WA: Western Asia; LA: Latin America. Supplementary Dataset 3 lists countries in each aggregated region.

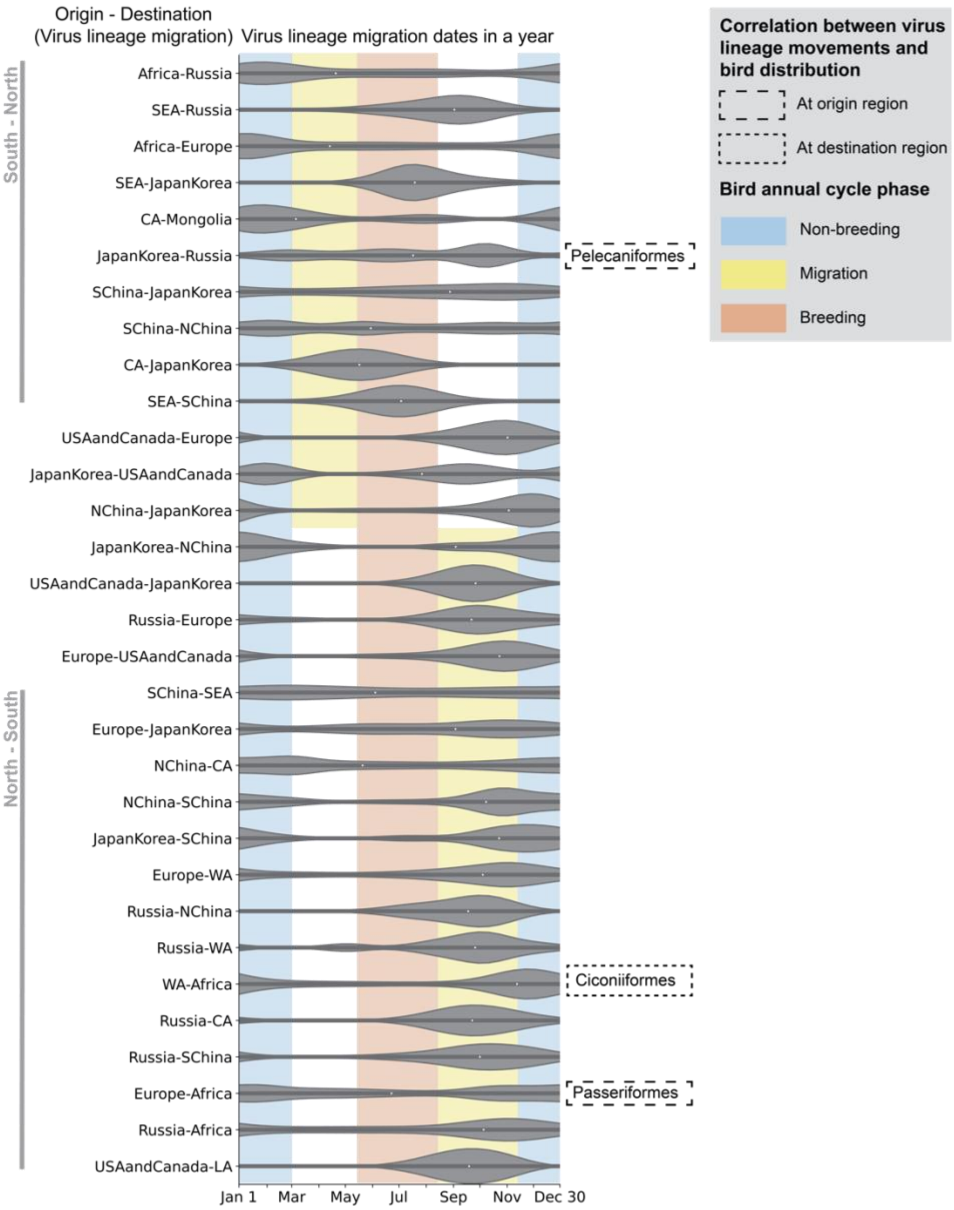

Figure S7. Probability density distribution of the virus lineage migration throughout the year, between locations summarized from the discrete trait phylogeography of HPAIV H5 clade 2.3.4.4 (from 2018 to 2023) and the Markov jump counts. X axis: Virus lineage migration dates in a year; Y axis: origin region - destination region of the virus lineage migration. The width of the violins represents the virus lineage migration probability density. Non-breeding (blue), migration (yellow) and breeding (red) bird annual cycle phases in general are shown in the south-north migration direction and in the north-south migration direction. Boxes around bird orders show the statistically significant correlation of virus lineage movements and bird order distribution at origin, destination or both regions. Block bootstrapping was used to calculate confidence intervals and two-tailed p values. See results in Table S3 and methods in Methods 4.4. Abbreviations of locations: NChina (North China), SChina (South China), SEA (South-East Asia), CA (Central Asia), WA (Western Asia), LA (Latin America). Supplementary Dataset 3 lists countries in each aggregated region.

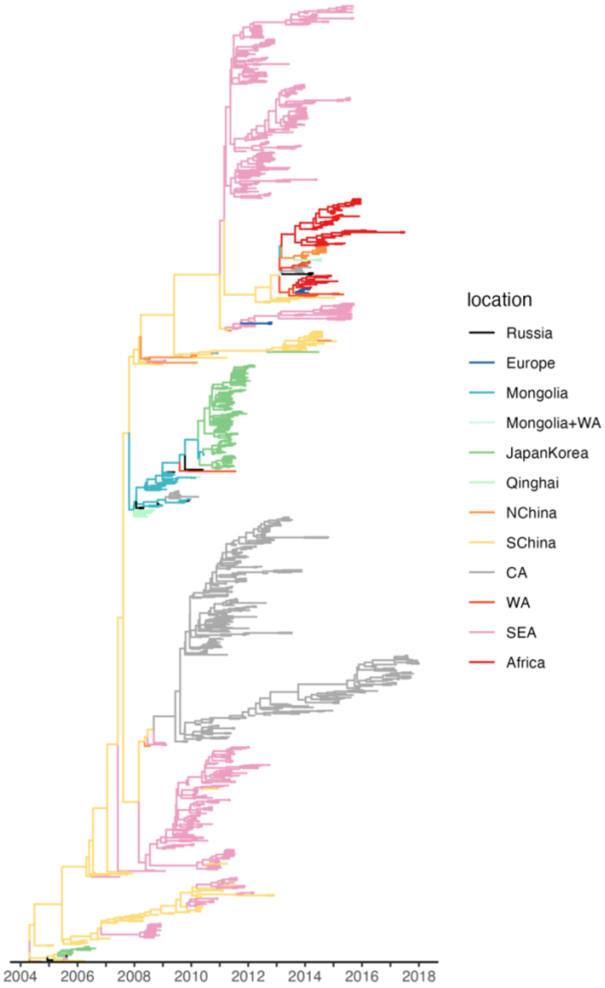

Figure S8. Phylogeographic reconstructions for clade 2.3.2.1 shown as time-scaled maximum clade credibility (MCC) trees with location annotations to summarize the reconstructions. Abbreviations of the locations are same as in Figure 1 and S7.

References

1. Pinzon, J. E. & Tucker, C. J. A Non-Stationary 1981–2012 AVHRR NDVI3g Time Series. *Remote Sensing* **6**, 6929–6960 (2014).
2. Tuanmu, M.-N. & Jetz, W. A global 1-km consensus land-cover product for biodiversity and ecosystem modelling. *Global Ecology and Biogeography* **23**, 1031–1045 (2014).
3. Fick, S. E. & Hijmans, R. J. WorldClim 2: new 1-km spatial resolution climate surfaces for global land areas. *International Journal of Climatology* **37**, 4302–4315 (2017).
4. Farr, T. G. & Kobrick, M. Shuttle radar topography mission produces a wealth of data. *Eos, Transactions American Geophysical Union* **81**, 583–585 (2000).
5. Farr, T. G. *et al.* The Shuttle Radar Topography Mission. *Rev. Geophys.* **45**, RG2004 (2007).
6. OpenTopography. Shuttle Radar Topography Mission (SRTM) Global. (2013) doi:10.5069/G9445JDF.
7. Rosen, P. A. *et al.* Synthetic aperture radar interferometry. *Proceedings of the IEEE* **88**, 333–382 (2000).
8. Kobrick, M. On the Toes of Giants - How SRTM was Born. *Photogramm. Eng. Remote Sens.* 206–210 (2006).
