# Supplementary material for: Synchrony of Bird Migration with Global Dispersal of Avian Influenza Reveals Exposed Bird Orders
Source: Nat Commun. 2024 Feb 6;15:1126. doi: 10.1038/s41467-024-45462-1 (PMC10847442; doi:10.1038/s41467-024-45462-1)
Supplement: Supplementary file 3 — Description of Additional Supplementary Files [file 41467_2024_45462_MOESM3_ESM.pdf]

Description of Additional Supplementary Files

File Name: Supplementary Data 1
Description: Virus genomic sequence metadata

File Name: Supplementary Data 2
Description: Metadata of Movebank studies used in quantitative analysis (Figure 3)

File Name: Supplementary Data 3
Description: The dictionary of country - aggregated location
