# Supplementary material for: Synchrony of Bird Migration with Global Dispersal of Avian Influenza Reveals Exposed Bird Orders
Source: Nat Commun. 2024 Feb 6;15:1126. doi: 10.1038/s41467-024-45462-1 (PMC10847442; doi:10.1038/s41467-024-45462-1)
Supplement: Supplementary file 7 — Reporting Summary [file 41467_2024_45462_MOESM7_ESM.pdf]

nature portfolio

Corresponding author(s): Qiqi Yang, Huaiyu Tian, Bryan T. Grenfell

Last updated by author(s): Jan/10/24

Reporting Summary

Nature Portfolio wishes to improve the reproducibility of the work that we publish. This form provides structure for consistency and transparency in reporting. For further information on Nature Portfolio policies, see our Editorial Policies and the Editorial Policy Checklist.

Statistics

For all statistical analyses, confirm that the following items are present in the figure legend, table legend, main text, or Methods section.

n/a	Confirmed	
☐	☒	The exact sample size (*n*) for each experimental group/condition, given as a discrete number and unit of measurement
☐	☒	A statement on whether measurements were taken from distinct samples or whether the same sample was measured repeatedly
☐	☒	The statistical test(s) used AND whether they are one- or two-sided<br>*Only common tests should be described solely by name; describe more complex techniques in the Methods section.*
☐	☒	A description of all covariates tested
☐	☒	A description of any assumptions or corrections, such as tests of normality and adjustment for multiple comparisons
☐	☒	A full description of the statistical parameters including central tendency (e.g. means) or other basic estimates (e.g. regression coefficient) AND variation (e.g. standard deviation) or associated estimates of uncertainty (e.g. confidence intervals)
☐	☒	For null hypothesis testing, the test statistic (e.g. *F*, *t*, *r*) with confidence intervals, effect sizes, degrees of freedom and *P* value noted<br>*Give P values as exact values whenever suitable.*
☐	☒	For Bayesian analysis, information on the choice of priors and Markov chain Monte Carlo settings
☒	☐	For hierarchical and complex designs, identification of the appropriate level for tests and full reporting of outcomes
☐	☒	Estimates of effect sizes (e.g. Cohen's *d*, Pearson's *r*), indicating how they were calculated

Our web collection on statistics for biologists contains articles on many of the points above.

Software and code

Policy information about availability of computer code

Data collection	Custom software (version 1.0.0): https://github.com/kikiyang/HPAI_Bird_world
Data analysis	Custom software (version 1.0.0): https://github.com/kikiyang/HPAI_Bird_world

For manuscripts utilizing custom algorithms or software that are central to the research but not yet described in published literature, software must be made available to editors and reviewers. We strongly encourage code deposition in a community repository (e.g. GitHub). See the Nature Portfolio guidelines for submitting code & software for further information.

Data

Policy information about availability of data

All manuscripts must include a data availability statement. This statement should provide the following information, where applicable:

- Accession codes, unique identifiers, or web links for publicly available datasets
- A description of any restrictions on data availability
- For clinical datasets or third party data, please ensure that the statement adheres to our policy

We provide Movebank Study ID (unique searchable identifier) and relevant metadata information for bird tracking data available on Movebank database (movebank.org) in Supplementary Dataset 2. We provide accession ID and relevant metadata for virus genomic data available on GISAID database (gisaid.org) in Supplementary Dataset 1. Live poultry trade data are available on the United Nations Comtrade Database (comtrade.un.org/data/). Live poultry trade data within China and the country-level poultry population size data used in phylogeographic analyses are available on the GitHub Repository. The environmental variable data

used in the species distribution model are publicly available on WorldClim (www.worldclim.com/version2), NASA ARC ECOCAST GIMMS NDVI3g v1p0: Version 1.0. (iridl.ldeo.columbia.edu/SOURCES/.NASA/.ARC/.ECOCAST/.GIMMS/.NDVI3g/.v1p0/index.html?Set-Language=en), Global 1-km Consensus Land Cover (www.earthenv.org/landcover) and LP DAVV and Data Products (lpdaac.usgs.gov/about/citing_lp_daac_and_data).

Research involving human participants, their data, or biological material

Policy information about studies with human participants or human data. See also policy information about sex, gender (identity/presentation), and sexual orientation and race, ethnicity and racism.

Reporting on sex and gender	N/A
Reporting on race, ethnicity, or other socially relevant groupings	N/A
Population characteristics	N/A
Recruitment	N/A
Ethics oversight	N/A

Note that full information on the approval of the study protocol must also be provided in the manuscript.

Field-specific reporting

Please select the one below that is the best fit for your research. If you are not sure, read the appropriate sections before making your selection.

☐ Life sciences ☐ Behavioural & social sciences ☒ Ecological, evolutionary & environmental sciences

For a reference copy of the document with all sections, see nature.com/documents/nr-reporting-summary-flat.pdf

Ecological, evolutionary & environmental sciences study design

All studies must disclose on these points even when the disclosure is negative.

Study description	The study aims to understand what bird orders are exposed to highly pathogenic avian influenza H5 and where by integrating both bird migration and virus genomic data. We use virus genomic data of highly pathogenic avian influenza H5 clade 2.3.4.4 and 2.3.2.1 to infer the evolutionary history and the phylogeography of viruses. We also utilize wild bird tracking data of various species at global level to infer 1) a qualitative conceptual network of bird migration, and 2) species distribution at order level. We quantified the contribution of bird migration network to the virus lineage movements between regions by applying the generalized linear model extended Bayesian phylogeography inference with the 1000 empirical trees as the input. We quantified the synchrony of bird distribution at regions with virus lineage movements from or to the region by applying block bootstrapping method to compute mean correlation with confidence intervals and two-tailed p values.
Research sample	We acquired samples from existing datasets:<br>1. Bird tracking data from Movebank database to represent the global migratory bird population. The samples include 53 studies, 3542 individuals (class: Ave), 10 orders and 95 species. The samples are collected by various research groups, and by various sensors, including Global Position System (GPS), Argos, bird ring, radio transmitter, solar geo-locator, and natural mark.<br>2. Virus genomic data (HA gene) from GISAID database to represent the global populations of highly pathogenic avian influenza H5 clade 2.3.4.4 and 2.3.2.1. The samples are collected and sequenced by various research groups.
Sampling strategy	1. Due to low availability of bird tracking data in the tracking community, to maximize the data coverage in species and space, we obtained as many Movebank studies as possible which are accessible for scientific reuse.<br>2. For early virus genomic data, due to limited sampling intensity, especially in wild birds, to maximize the genetic diversity of the viruses in analysis, we obtained all samples on GISAID. For most recent virus genomic data of clade 2.3.4.4, due to the hugely increased sampling intensity, after we accessed all samples from GISAID, we randomly down-sampled the over-represented locations (with sequences more than 250) to 262 for each location to get a similar size of sequences across locations and to get a similar size of total sequences as previous clade 2.3.4.4.
Data collection	Qiqi Yang downloaded the data from publicly available databases: GISAID (www.gisaid.org) and Movebank (www.movebank.org).
Timing and spatial scale	Virus genomic data (HA gene): clade 2.3.4.4, global level, from 2009-12 to 2023-07; clade 2.3.2.1, global level, from 2007-05 to 2017-12; Bird tracking data: global level, from 1966-05 to 2021-10 (different bird tracking studies have varying timing and spatial scale)
Data exclusions	1. Clade 2.3.4.4 (2018-2023) virus genomic data were randomly down-sampled. The sequences at over-represented locations were randomly excluded to get a similar size of sequences across locations and to get a similar size of total sequences as previous clade 2.3.4.4.<br>2. We excluded the bird tracking data of Cuculiformes, Caprimulgiformes, Strigiformes, Columbiformes, Phoenicopteriformes, Piciformes, Sphenisciformes, and Procellariiformes from Movebank bird tracking dataset, given their paucity and geographically

	restricted distribution, when modeling the species distribution.<br>3. In other datasets, no data were excluded.
Reproducibility	1. The statistical analyses were repeated to get credible levels or confidence levels and therefore the reproducibility should be verified.<br>2. All the data and scripts are provided on the GitHub repository for reproducibility.
Randomization	1. We randomly down-sampled over-represented locations (with sequences more than 250) to 262 for each location to get a similar size of sequences across locations and to get a similar size of total sequences as previous clade 2.3.4.4.<br>2. We conducted bootstrapping when computing correlation between bird distribution and virus lineage movements.
Blinding	1. We blinded ourselves to data provider names and institutions when we accessed the data from GISAID.<br>2. We were not able to blind ourselves to data provider names on Movebank because we had to contact them for permission on reuse the data.

Did the study involve field work? ☐ Yes ☒ No

Reporting for specific materials, systems and methods

We require information from authors about some types of materials, experimental systems and methods used in many studies. Here, indicate whether each material, system or method listed is relevant to your study. If you are not sure if a list item applies to your research, read the appropriate section before selecting a response.

Materials & experimental systems

n/a	Involved in the study
☒	☐ Antibodies
☒	☐ Eukaryotic cell lines
☒	☐ Palaeontology and archaeology
☒	☐ Animals and other organisms
☒	☐ Clinical data
☒	☐ Dual use research of concern
☒	☐ Plants

Methods

n/a	Involved in the study
☒	☐ ChIP-seq
☒	☐ Flow cytometry
☒	☐ MRI-based neuroimaging
